# Association between *MTHFR* C677T/A1298C and susceptibility to autism spectrum disorders: a meta-analysis

**DOI:** 10.1186/s12887-020-02330-3

**Published:** 2020-09-24

**Authors:** Yan Li, Shuang Qiu, Jikang Shi, Yanbo Guo, Zhijun Li, Yi Cheng, Yawen Liu

**Affiliations:** 1 Department of Epidemiology and Biostatistics, School of Public Health, Jilin University, Changchun, 130021 China; 2 Institute of Translational Medicine, the First Hospital of Jilin University, Changchun, 130021 China

**Keywords:** Methylenetetrahydrofolate reductase, Autism spectrum disorder, Single nucleotide polymorphisms, Genetic models, Meta-analysis

## Abstract

**Background:**

Autism spectrum disorder (ASD) is becoming increasingly prevalent of late. Methylenetetrahydrofolate reductase (MTHFR) has a significant role in folate metabolism. Owing to the inconsistencies and inconclusiveness on the association between *MTHFR* single nucleotide polymorphism (SNP) and ASD susceptibilities, a meta-analysis was conducted to settle the inconsistencies.

**Methods:**

For this meta-analysis, a total of 15 manuscripts published up to January 26, 2020, were selected from PubMed, Google Scholar, Medline, WangFang, and CNKI databases using search terms “MTHFR” OR “methylenetetrahydrofolate reductase” AND “ASD” OR “Autism Spectrum Disorders” OR “Autism” AND “polymorphism” OR “susceptibility” OR “C677T” OR “A1298C”.

**Results:**

The findings of the meta-analysis indicated that *MTHFR* C677T polymorphism is remarkably associated with ASD in the five genetic models, viz., allelic, dominant, recessive, heterozygote, and homozygote. However, the *MTHFR* A1298C polymorphism was not found to be significantly related to ASD in the five genetic models. Subgroup analyses revealed significant associations of ASD with the *MTHFR* (C677T and A1298C) polymorphism. Sensitivity analysis showed that this meta-analysis was stable and reliable. No publication bias was identified in the associations between *MTHFR*C677T polymorphisms and ASD in the five genetic models, except for the one with regard to the associations between *MTHFR*A1298C polymorphisms and ASD in the five genetic models.

**Conclusion:**

This meta-analysis showed that *MTHFR* C677T polymorphism is a susceptibility factor for ASD, and *MTHFR* A1298C polymorphism is not associated with ASD susceptibility.

## Background

Autism spectrum disorder (ASD) is one of the complex neurodevelopmental disorders, which has been increasingly recognized as a public health issue [1]. It affects 9‰ of the entire population of children, and the estimated ratio between male and female (M:F) children is 4:1 [2]. The prevalence rates of ASD in terms of percentages are approximately 1.52‰ in the Middle East [2–5], 14.7‰ in the USA [6, 7], 1.66‰ in China [8], and 6‰ in Australia [1, 9].

The distinguishing features of ASD include a set of behavioral phenotypes such as social communication deficits, restrictive and repetitive behaviors [10, 11], and worsened quality of life and family functioning for children with ASD and their parents [12]. Brain and nervous system dysfunctions are indicated in ASD [13], which occur as a result of pathophysiological and environmental factors. Folate/homocysteine (Hcy) levels act as a risk factor in ASD [14, 15], indicating the involvement of methylenetetrahydrofolate reductase (MTHFR) in ASD. Therefore, MTHFR has been the focal point of investigation on ASD, as inheritance validates the pathophysiological mechanism of ASD [16–18].

*MTHFR* locus has been mapped to chromosome1 (1p36.3) [19]. Conversion of 5, 10-methylenetetrahydrofolate to 5-methylenetetrahydrofolate is performed by MTHFR, which regulates the intracellular levels of folate and Hcy [15, 20]. Single nucleotide polymorphisms (C677T and A1298C) are associated with the decline in MTHFR activity [21, 22], which is, in turn, correlated with Folate/Hcy levels [23, 24]. Homocysteinemia and low plasma folate are found in individuals with C677T and A1298C alleles [22, 25]. A reduction of approximately 50% ~ 60% in the MTHFR activity is correlated with compound heterozygosity for both C677T and A1298C [19, 22, 26–28]. A decline in the enzymatic activity to 35% ~ 70% in homozygotes T is linked to C677T polymorphism in *MTHFR* [29]. Generally, when compared to C677T mutation, A1298C mutation feebly affects MTHFR activity and Hcy and folate levels [25, 30].

Correlations between single nucleotide polymorphisms (C677T and A1298C) and susceptibility to ASD are still debatable. A correlation between *MTHFR* C677T polymorphism and a higher susceptibility to ASD has been reported by Boris et al. [22] among Caucasian children [27]. Guo et al. [31] evidenced that *MTHFR* C677T polymorphism is a risk factor for ASD among Chinese Han children [31]. El-baz et al. [32] recognized a significant correlation between *MTHFR* C677T polymorphisms and ASD among Egyptian children [32]. Nonetheless, Dos Santos et al. [28] found no correlation between *MTHFR* C677T polymorphism and ASD [28]. Studies by Khalil et al. [33] and El-baz et al. [32, 34] describe *MTHFR* A1298C polymorphism to represent a risk factor in correlation with ASD among Egyptian children. On the contrary, Mohammad et al. [35] evidenced that *MTHFR* A1298C polymorphism variant allele has no link with any independent risk of ASD [35]. In this meta-analysis, updated articles were gathered [26, 32, 36] to authenticate correlations between *MTHFR* polymorphism (C677T/A1298C) and susceptibility to ASD.

## Methods

### Search strategy and identification of studies

Scientific literature published before January 26, 2020, in PubMed, Embase, Web of Science, Medline, WanFang datebase, and CNKI database were searched using specific search terms (Supplement file 1). The equivalent Chinese terms were used in the Chinese databases. Moreover, we retrieved related articles from the selected literature references to replenish data that had not been identified in the initial search. All full-text literature were scrutinized to determine whether the papers to be included.

### Selection criteria

The following criteria had to be satisfied by the studies to be incorporated in this meta-analysis: (1) Original studies on the correlation between *MTHFR* polymorphism (C677T/A1298C) and ASD; (2) Cohort or case-control designs; (3) All genotype frequency information is available; (4) Diagnostic criteria of ASD described in the *Diagnostic and Statistical Manual of Mental Disorders* (*4th* or *5th edition*) [37, 38], and/or Childhood Autism Rating Scale [39]. Certain earlier papers referred to the *Manual of Mental Disorders* (*3rd edition*) [40]. The exclusion criteria comprised the following: (1) Researches on the correlation between *MTHFR* polymorphism (C677T/A1298C) and ASD that are not original; (2) Studies that lack data and complete information; (3) Replicated studies; (4) Review studies.

### Data extraction

Two investigators, namely, Yan Li and Shuang Qiu, extracted all the relevant data with the help of a standardized protocol and data collection form. From every qualified study, data such as the name of the first author, year of publication, country, study population (ethnicity), study design, the definition of ASD, sample size of cases and controls, genotyping method, genotype information, and allele frequencies were gathered and documented. Disparities in the study selection were resolved through discussion or consensus with the third investigator (Yawen Liu). The corresponding authors of articles with missing data were emailed for the required data.

### Statistical analysis

Odds ratio (*OR*) and 95% confidence intervals (*CI*) were deduced to analyse how strongly *MTHFR* (C677T/A1298C) polymorphism and the risk of ASD were correlated in the five genetic models, viz., allelic, dominant, recessive, heterozygote, and homozygote. Heterogeneity among studies was assessed through *Q*-test and *I*^*2*^. Random effects model (DerSimonian-Laird methods) [41] was selected to pool data and in case of substantial heterogeneity (*Ph* < 0.05 and *I*^*2*^ > 50%); else, fixed effect model (Mantel-Haenszel methods) [42] was chosen. Furthermore, subgroup analyses were stratified according to the state with mandatory fortification of folate, population, sample source, and Hardy-Weinberg equilibrium (HWE). The included studies were tested for HWE in the control group utilizing Chi-square tests. Besides, the stability of the results was tested by performing a sensitivity analysis with the sequential omission of each study. To evaluate the potential publication bias in this meta-analysis, Begg’s funnel plot and Egger’s test were conducted. Stata version 12.0 (StataCorp LP, College Station, TX, USA) was used to evaluate all analyses, and *p* < 0.05 was considered to be statistically significant.

## Results

### Overall results

**Fig. 1:**
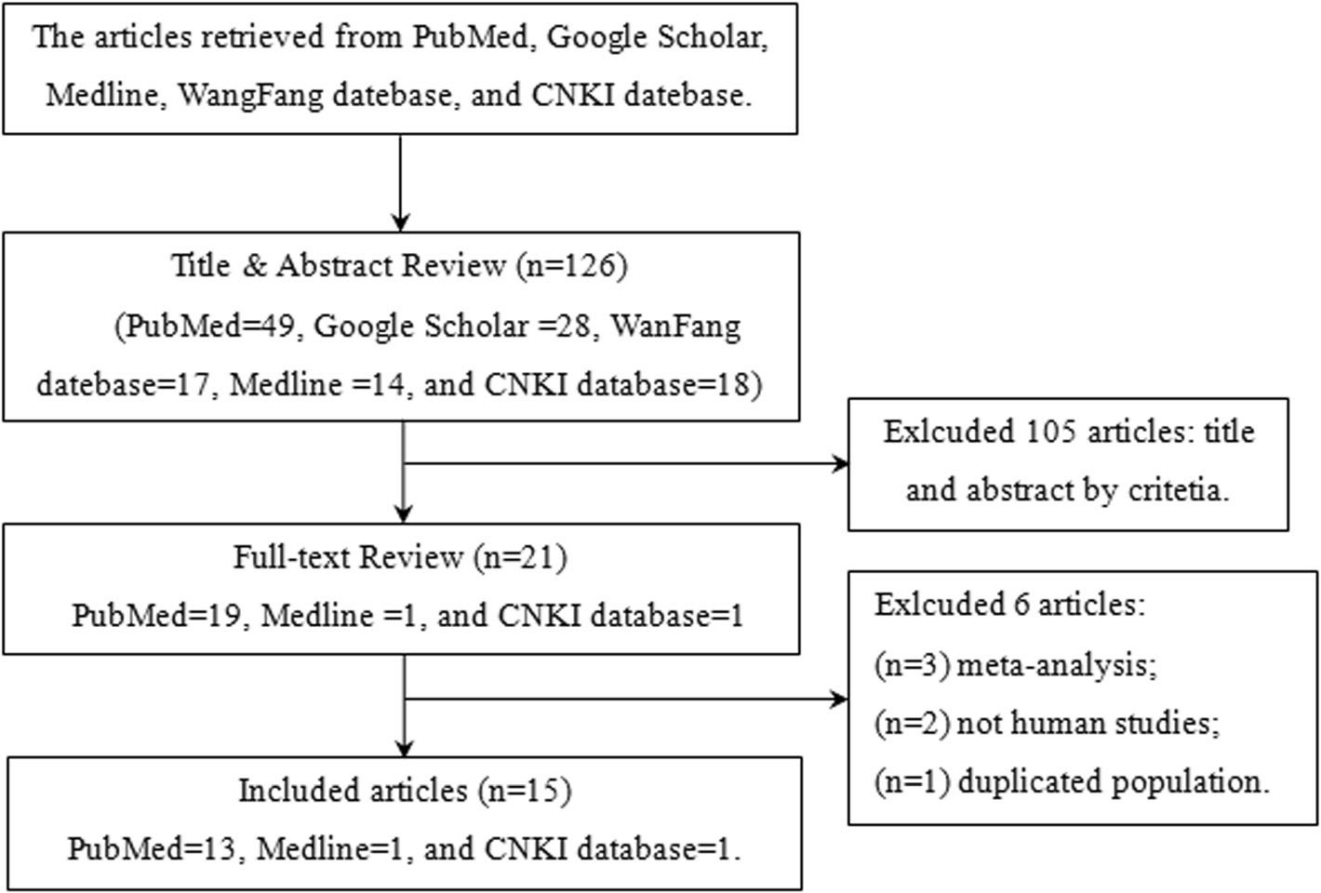
Flowchart of this meta-analysis

Upon literature search and critical screening, about 15 studies from 125 articles were included in this meta-analysis, as already discussed in the Methods section (Fig. 1). A total of 2609 cases and 7496 controls were enrolled from the 15 articles published on the correlation between *MTHFR* C677T polymorphism and ASD susceptibility. Of those, only nine articles that included 1961 cases and 1652 controls qualified for the evaluation of the link between *MTHFR* A1298C and ASD as per the selection criteria. The characteristics of each primary study are summarized and presented in Tables 1 and 2.

**Table 1:** Characteristics of included studies for *MTHFR* C677T polymorphism

Author, year	Quality Score	Country	Ethnicity	Case				Control				Sample source	Folate	HWE
				N	CC	CT	TT	N	CC	CT	TT			
Boris et al. 2004 [22]	6	USA	Caucasian	168	35	94	39	5389	2570	2213	606	Hospital-based	YES	0
James et al. 2006 [43]	7	USA	Caucasian	356	134	176	46	205	93	90	22	Hospital-based	YES	0.974
Mohammad et al. 2009 [35]	7	USA	Asian	138	98	35	5	138	120	18	0	Population-based	NO	0.412
Pasca et al. 2009 [27]	8	Romania	Caucasian	39	21	14	4	80	46	28	6	Population-based	NO	0.551
dos Santos et al. 2010 [28]	7	Brazil	Caucasian	151	60	68	23	100	45	41	14	Hospital-based	YES	0.353
Liu et al. 2011 [44]	7	Canada	Caucasian	205	68	98	39	384	177	166	41	Population-based	YES	0.823
Liu et al. 2011 [44]	7	Canada	Caucasian	400	167	179	54	384	177	166	41	Population-based	YES	0.823
Schmidt et al. 2011 [45]	8	USA	Caucasian	294	128	133	33	180	74	77	29	Population-based	YES	0.241
Guo et al. 2012 [31]	7	China	Asian	186	79	77	30	186	87	83	16	Population-based	NO	0.542
Divyakolu et al. 2013 [46]	6	India	Asian	50	27	22	1	50	42	8	0	Hospital-based	NO	0.539
Park et al. 2014 [47]	7	Korea	Asian	249	76	136	37	423	139	204	80	Hospital-based	NO	0.737
Sener et al. 2014 [48]	9	Turkey	Caucasian	98	44	51	3	70	37	33	0	Population-based	NO	0.009
Shawky et al. 2014 [46]	6	Egypt	Caucasian	20	7	10	3	22	16	6	0	Hospital-based	NO	0.459
Meguid et al. 2015 [49]	8	Egypt	Caucasian	24	11	11	2	30	20	8	2	Population-based	NO	0.361
El-baz et al. 2017 [32]	6	Egypt	Caucasian	31	12	15	4	39	35	4	0	Hospital-based	YES	0.735
Zhao et al. 2013 [36]	9	China	Asian	200	91	59	50	200	144	39	17	Hospital-based	NO	0

**Table 2:** Characteristics of included studies for *MTHFR* A1298C polymorphism

Author, year	Quality Score	Country	Ethnicity	Case				Control				Sample source	Folate	HWE
				N	AA	AC	CC	N	AA	AC	CC			
Boris et al. 2004 [22]	6	USA	Caucasian	168	93	65	10	159	70	75	14	Hospital-based	YES	0
James et al. 2006 [43]	7	USA	Caucasian	356	175	147	34	204	103	77	24	Hospital-based	YES	0.974
Mohammad et al. 2009 [35]	7	USA	Asian	138	35	59	44	138	48	32	58	Population-based	NO	0.412
Liu et al. 2011 [44]	8	Canada	Caucasian	205	109	81	15	382	170	175	37	Population-based	YES	0.823
Liu et al. 2011 [44]	7	Canada	Caucasian	307	134	133	40	382	170	175	37	Population-based	YES	0.823
Schmidt et al. 2011 [45]	8	USA	Caucasian	296	160	117	19	177	89	76	12	Population-based	YES	0.241
Park et al. 2014 [47]	6	Korea	Asian	236	147	75	14	323	198	114	11	Hospital-based	NO	0.737
Meguid et al. 2015 [49]	8	Egypt	Caucasian	24	0	23	1	30	12	16	2	Population-based	NO	0.361
El-baz et al. 2017 [32]	6	Egypt	Caucasian	31	7	13	11	39	31	7	1	Hospital-based	YES	0.451
Zhao et al. 2013 [36]	9	China	Asian	200	144	19	37	200	166	21	13	Hospital-based	NO	0

### Association between MTHFR C677T polymorphism and ASD

**Fig. 2:**
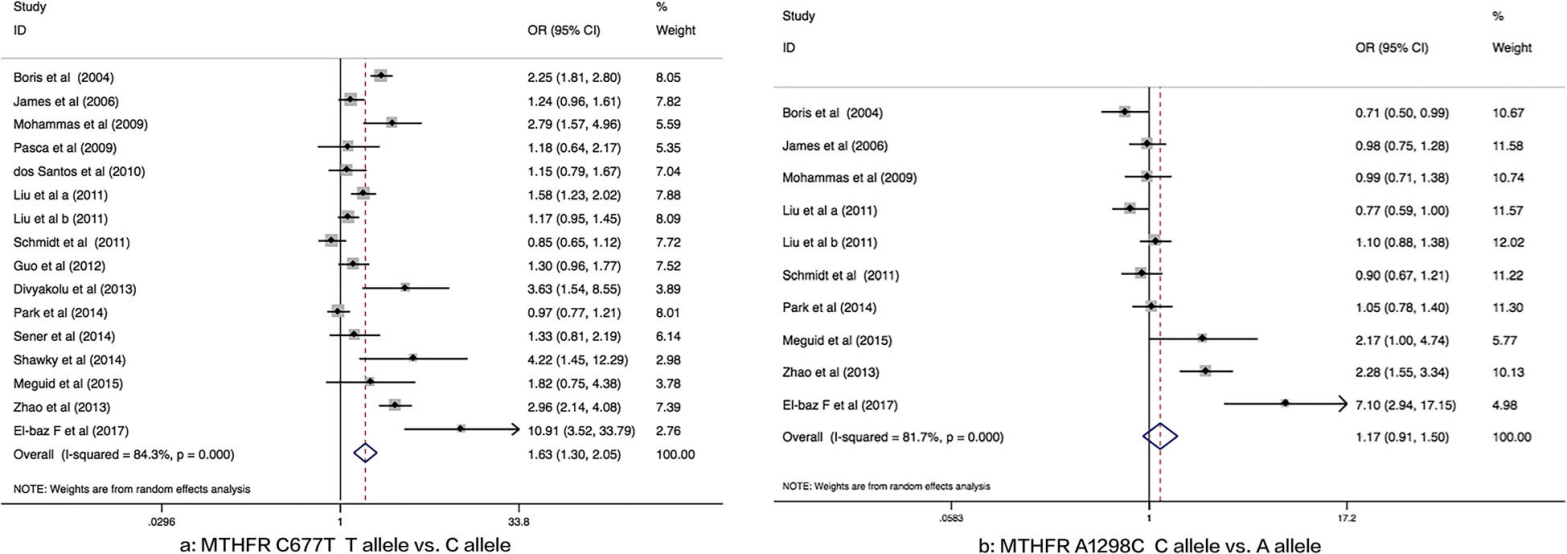
Association between *MTHFR* (C677T and A1298C) polymorphism and ASD susceptibility

Random effect model (*P*_*h*_ < 0.05 or *I*^*2*^ > 50%) was used, and *MTHFR* C677T polymorphism was found to be remarkably linked to ASD susceptibility in allelic (T vs C: *OR* = 1.63, 95% *CI* = 1.30–2.05, *p* < 0.05), heterozygote (CT vs CC: *OR =* 1.66, 95% *CI* = 1.31–2.11, *p* < 0.05), homozygote (TT vs CC: *OR* = 2.03, 95% *CI* = 1.33–3.09, *p* < 0.05), dominant (TT + CT vs CC: *OR* = 1.82, 95% *CI* = 1.39–2.37, *p* < 0.05), and recessive models (TT vs CT + CC: *OR* = 1.59, 95% *CI* = 1.14–2.22, *p* < 0.05; Table 3, Fig. 2a).

**Table 3:** Meta-analysis between *MTHFR* C677T polymorphism and ASD risk under genetic models

Genetic Models	Fixed/ Random effect *OR*(95%CI)	Heterogeneity *P*	*I*^*2*^(%)	Publication Bias *P* of Egger’s/Begg test
Allele Contrast (T vs C)	1.63 (1.30–2.05)^b*^	0.000	84.3	0.029/0.017
Mandatory fortification with folate				
Yes	1.32 (1.00–1.75)^b^	0.000	86.2	0.441/0.707
No	2.08 (1.40–3.08)^b*^	0.000	84.4	0.044/0.032
Population				
Asian	1.95 (1.14–3.33)^b*^	0.000	90.3	0.178/0.221
Caucasian	1.51 (1.17–1.95)^b*^	0.000	81.5	0.130/0.087
Sample source				
Hospital-based	2.10 (1.34–3.14)^b*^	0.000	89.6	0.062/0.174
Population-based	1.33 (1.11–1.65)^b*^	0.006	64.3	0.267/0.386
HWE				
Yes	1.46 (1.18–1.81)^b*^	0.000	76.0	0.005/0.006
No	2.17 (1.52–3.10)^b*^	0.030	71.4	0.779/1.000
Heterozygote (CT vs CC)	1.66 (1.31–2.11)^b*^	0.000	69.2	0.017/0.008
Mandatory fortification with folate				
Yes	1.45 (1.05–2.00)^b*^	0.001	76.1	0.784/0.707
No	1.95 (1.34–2.82)^b*^	0.002	66.4	0.031/0.020
Population				
Asian	1.80 (1.15–2.80)^b*^	0.005	72.7	0.044/0.221
Caucasian	1.62 (1.20–2.18)^b*^	0.000	70.4	0.098/0.029
Sample source				
Hospital-based	2.23 (1.48–3.35)^b*^	0.000	76.3	0.048/0.108
Population-based	1.26 (1.07–1.48)^a*^	0.249	22.6	0.191/0.266
HWE				
Yes	1.49 (1.18–1.87)^b*^	0.005	57.9	0.007/0.009
No	2.24 (1.40–3.58)^b*^	0.064	63.6	0.001/0.296
Homozygote (TT vs CC)	2.03 (1.33–3.09)^b*^	0.000	74.6	0.048/0.053
Mandatory fortification with folate				
Yes	1.66 (0.94–2.94)^b^	0.000	84.7	0.355/0.700
No	2.78 (1.35–5.73)^b*^	0.001	66.5	0.044/0.074
Population				
Asian	2.45 (0.95–6.31)^b^	0.000	81.2	0.286/0.806
Caucasian	1.92 (1.16–3.16)^b*^	0.000	73.7	0.147/0.119
Sample source				
Hospital-based	2.54 (1.26–5.16)^b*^	0.000	82.5	0.142/0.536
Population-based	1.61 (1.01–2.58)^b*^	0.031	54.7	0.122/0.266
HWE				
Yes	1.50 (1.05–2.13)^b*^	0.012	53.4	0.006/0.012
No	4.72 (3.26–6.84)^a*^	0.988	0.0	0.291/1.000
Dominant (TT + CT vs CC)	1.82 (1.39–2.37)^b*^	0.000	78.6	0.021/0.010
Mandatory fortification with folate				
Yes	1.49 (1.04–2.15)^b*^	0.000	83.3	0.775/0.707
No	2.22 (1.46–3.36)^b*^	0.000	76.3	0.051/0.049
Population				
Asian	2.03 (1.21–3.42)^b*^	0.000	82.7	0.164/0.221
Caucasian	1.73 (1.25–2.41)^b*^	0.000	78.4	0.089/0.029
Sample source				
Hospital-based	2.51 (1.57–4.02)^b*^	0.000	84.6	0.050/0.108
Population-based	1.32 (1.13–1.54)^a*^	0.066	47.2	0.253/0.266
HWE				
Yes	1.59 (1.23–2.04)^b*^	0.000	68.3	0.008/0.003
No	2.59 (1.60–4.18)^b*^	0.038	69.5	0.016/0.296
Recessive (TT vs CT + CC)	1.59 (1.14–2.22)^b*^	0.000	65.6	0.033/0.053
Mandatory fortification with folate				
Yes	1.37 (0.93–2.00)^b^	0.003	72.3	0.114/0.707
No	2.23 (1.13–4.38)^b*^	0.002	65.1	0.039/0.283
Population				
Asian	2.07 (0.84–5.10)^b*^	0.000	81.5	0.243/0.806
Caucasian	1.47 (1.04–2.07)^b*^	0.015	54.7	0.138/0.087
Sample source				
Hospital-based	1.76 (1.02–3.04)^b*^	0.000	76.0	0.155/0.386
Population-based	1.41 (1.11–1.80)^a*^	0.057	48.9	0.122/0.266
HWE				
Yes	1.23 (1.02–1.48)^a*^	0.025	48.7	0.006/0.033
No	2.79 (2.05–3.80)^a*^	0.459	0.0	0.489/1.000

*:*P* < 0.05

^a^Fixed effect

^b^Random effect

To further clarify the link between *MTHFR* polymorphisms and the risk of ASD, subgroup analysis was carried out. Firstly, no significant deviation of the correlation among the states with mandatory fortification of folate was recorded. *MTHFR* C677T polymorphism was not found to be linked to ASD susceptibility: allelic (T vs C: *OR* = 1.32, 95% *CI* = 1.00–1.75, *p* > 0.05), homozygote (TT vs CC: *OR* = 1.66, 95% *CI* = 0.94–2.94, *p* > 0.05), and recessive models (TT vs CT + CC: *OR* = 1.37, 95% *CI* = 0.93–2.00, *p* > 0.05). Nonetheless, it was observed to be associated with ASD susceptibility among the states without mandatory fortification of folate: allelic (T vs C: *OR* = 2.08, 95% *CI* = 1.40–3.08, *p* < 0.05), heterozygote (CT vs CC: *OR* = 1.95, 95% *CI* = 1.34–2.82, *p* < 0.05), homozygote (TT vs CC: *OR* = 2.78, 95% *CI* = 1.35–5.73, *p* < 0.05), dominant (TT + CT vs CC: *OR* = 2.22, 95% *CI* = 1.46–3.36, *p* < 0.05), and recessive models (TT vs CT + CC: *OR* = 2.23, 95% *CI* = 1.13–4.38, *p* < 0.05). Secondly, *MTHFR* C677T polymorphism was recorded to be correlated with ASD susceptibility in Caucasian population: allelic (T vs C: *OR* = 1.51, 95% *CI* = 1.17–1.95, *p* < 0.05), heterozygote (CT vs CC: *OR* = 1.62, 95% *CI* = 1.20–2.18, *p* < 0.05), homozygote (TT vs CC: *OR* = 1.92, 95% *CI* = 1.16–3.16, *p* < 0.05), and dominant models (TT + CT vs CC: *OR* = 1.73, 95% *CI* = 1.25–2.41, *p* < 0.05). Nonetheless, *MTHFR* C677T polymorphism was not found to be linked to ASD susceptibility among Asians: homozygote model (TT vs. CC: *OR* = 2.45, 95% *CI* = 0.95–6.31, *p* > 0.05). Thirdly, a hospital-based and population-based sample was adopted for this study. *MTHFR* C677T polymorphism was found to be linked with ASD susceptibility under five genetic models in hospital- and population-based samples, respectively (all *p* < 0.05). Fourthly, our results showed that *MTHFR* C677T polymorphism was consistent/inconsistent with HWE; however, it was significantly associated with ASD susceptibility under five genetic models (all *p* < 0.05) (Table 3).

### Association between MTHFR A1298C polymorphism and ASD

Random effect model (*P*_*h*_ < 0.05 or *I*^*2*^ ≥ 50%) was utilized, and no significant correlation between *MTHFR* A1298C polymorphism and ASD susceptibility in the five genetic models was identified (allelic, dominant, recessive, heterozygote, and homozygote; all *p* > 0.05; Table 4, Fig. 2b). As per the subgroup analyses, *MTHFR* A1298C polymorphism was found to be associated with ASD susceptibility among the states without mandatory fortification of folate: allelic model (C vs. A: *OR* = 1.84, 95% *CI* = 1.08–3.14, *p* < 0.05) and dominant model (CC + AC vs. AA: *OR* = 2.45, 95% *CI* = 1.16–5.15, *p* < 0.05). No significant correlation between *MTHFR* A1298C polymorphism and ASD susceptibility under the other genetic models in any subgroup was found (all *p* > 0.05) (Table 4).

**Table 4:** Meta-analysis of *MTHFR* A1298C polymorphism to ASD risk under the five genetic models

Genetic Models	Fixed/ Random effect *OR*(95%CI)	Heterogeneity *P*	*I*^*2*^(%)	Publication Bias *P* of Egger’s/Begg test
Allele Contrast (C vs A)	1.17 (0.91–1.50)^b^	0.000	81.7	0.210/0.010
Mandatory fortification with folate				
Yes	0.91 (0.81–1.03)^a^	0.153	40.3	0.086/0.098
No	1.84 (1.08–3.14)^b*^	0.002	86.0	0.086/0.095
Population				
Asian	1.31 (0.81–2.14)^b^	0.002	84.4	0.296/0.380
Caucasian	1.11 (0.82–1.49)^b^	0.000	80.9	0.548/0.045
Sample source				
Hospital-based	1.45 (0.88–2.39)^b^	0.000	89.5	0.221/0.021
Population-based	0.96 (0.84–1.10)^a^	0.074	53.0	0.204/0.462
HWE				
Yes	1.13 (0.84–1.52)^b^	0.000	80.9	0.368/0.043
No	1.25 (0.73–2.15)^b^	0.000	87.2	0.282/0.296
Heterozygote (AC vs AA)	1.11 (0.82–1.50)^b^	0.000	73.5	0.001/0.049
Mandatory fortification with folate				
Yes	0.87 (0.74–1.02)^a^	0.302	17.6	0.382/0.462
No	2.23 (0.98–5.09)^b^	0.000	82.7	0.026/0.086
Population				
Asian	1.29 (0.68–2.44)^b^	0.015	76.3	0.532/1.000
Caucasian	1.04 (0.72–1.50)^b^	0.001	74.7	0.002/0.230
Sample source				
Hospital-based	1.11 (0.71–1.74)^b^	0.004	74.0	0.090/0.462
Population-based	1.15 (0.72–1.86)^b^	0.001	78.4	0.009/0.221
HWE				
Yes	1.04 (0.73–1.50)^b^	0.001	74.6	0.001/0.133
No	1.28 (0.66–2.47)^b^	0.013	76.9	0.578/1.000
Homozygote (CC vs AA)	1.31 (0.82–2.09)^b^	0.000	72.0	0.025/0.152
Mandatory fortification with folate				
Yes	0.89 (0.67–1.18)^a^	0.260	24.2	0.139/0.462
No	2.98 (1.17–7.58)^b^	0.002	75.8	0.143/0.221
Population				
Asian	1.78 (0.88–3.62)^b^	0.041	68.8	0.811/1.000
Caucasian	1.11 (0.62–2.01)^b^	0.002	70.5	0.073/0.368
Sample source				
Hospital-based	1.87 (0.74–4.77)^b^	0.000	83.6	0.044/0.462
Population-based	1.02 (0.76–1.34)^a^	0.208	32.0	0.066/1.000
HWE				
Yes	1.27 (0.68–2.35)^b^	0.001	72.5	0.072/0.230
No	1.45 (0.65–3.24)^b^	0.014	76.7	0.966/1.000
Dominant (CC + AC vs AA)	1.19 (0.87–1.64)^b^	0.002	79.6	0.000/0.049
Mandatory fortification with folate				
Yes	0.87 (0.74–1.02)^a^	0.205	32.5	0.198/0.221
No	2.45 (1.16–5.15)^b*^	0.000	84.5	0.005/0.086
Population				
Asian	1.38 (0.89–2.14)^b^	0.054	65.8	0.291/1.000
Caucasian	1.13 (0.75–1.72)^b^	0.000	82.0	0.001/0.230
Sample source				
Hospital-based	1.43 (0.81–2.50)^b^	0.000	86.6	0.019/0.462
Population-based	1.03 (0.71–1.49)^b^	0.011	69.2	0.014/0.221
HWE				
Yes	1.14 (0.76–1.73)^b^	0.000	81.9	0.001/0.230
No	1.34 (0.80–2.23)^b^	0.023	73.4	0.306/1.000
Recessive (CC vs AC + AA)	1.17 (0.76–1.78)^b^	0.001	69.4	0.081/0.152
Mandatory fortification with folate				
Yes	0.94 (0.72–1.24)^a^	0.363	7.7	0.192/0.462
No	1.93 (0.70–1.25)^b^	0.000	82.6	0.240/0.806
Population				
Asian	1.52 (0.54–4.33)^b^	0.000	87.3	0.546/1.000
Caucasian	0.99 (0.64–1.55)^b^	0.486	52.8	0.174/0.368
Sample source				
Hospital-based	1.74 (0.76–3.99)^b^	0.000	80.3	0.063/0.462
Population-based	0.90 (0.69–1.19) ^a^	0.235	27.9	0.710/1.000
HWE				
Yes	1.12 (0.69–1.80)^b^	0.025	58.5	0.163/0.368
No	1.24 (0.46–3.36)^b^	0.001	86.6	0.676/1.000

*:*P* < 0.05

^a^Fixed effect

^b^Random effect

### Sensitivity analysis and publication bias

**Fig. 3:**
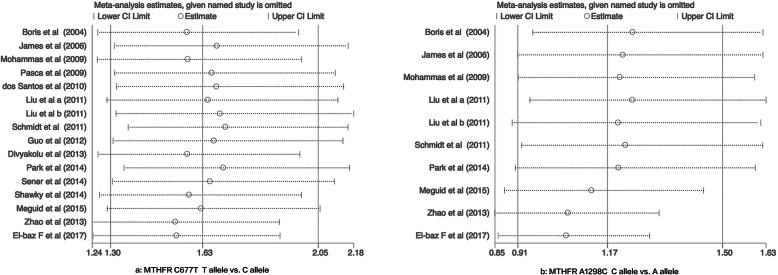
Sensitivity analysis between MTHFR (C677T and A1298C) polymorphism and ASD susceptibility

**Fig. 4:**
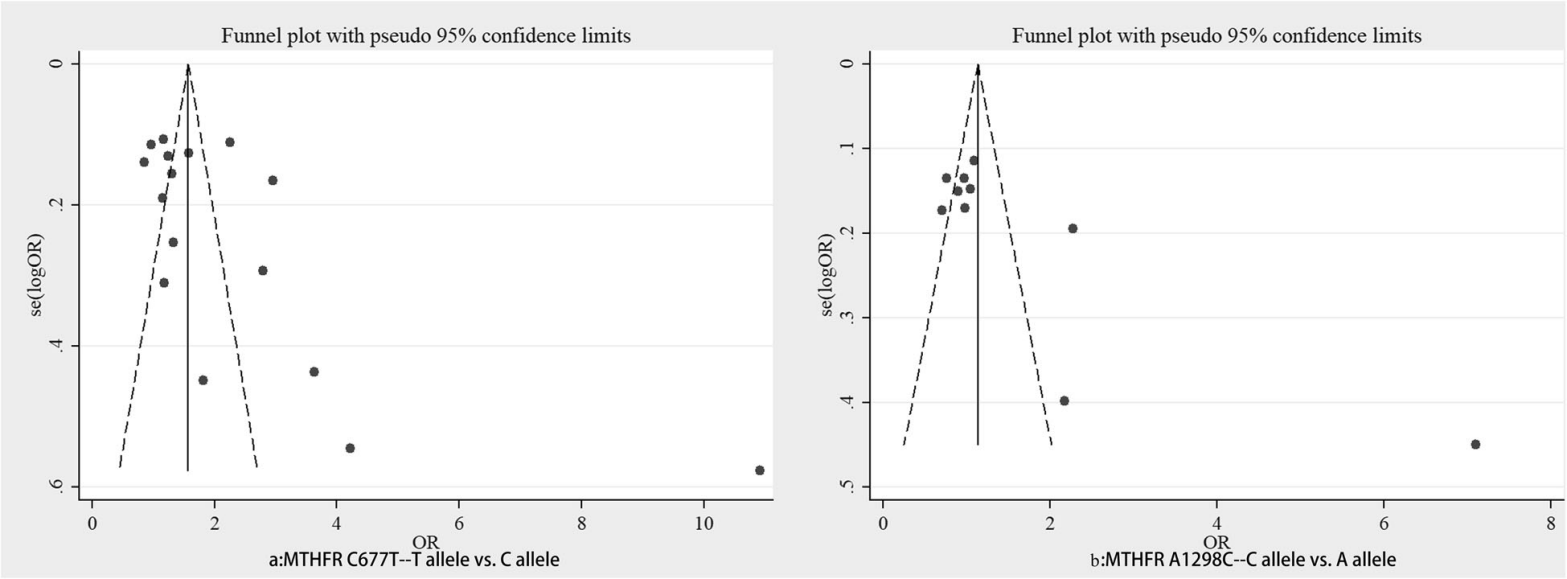
Publication bias between MTHFR (C677T and A1298C) polymorphism and ASD susceptibility

The stability of the findings was evaluated through sensitivity analysis conducted by sequentially omitting each study, demonstrating that this meta-analysis is relatively stable and credible (Fig. 3). To evaluate the publication bias, Begg’s funnel plot and Egger’s tests were carried out. No significant publication bias was detected in the correlation between *MTHFR* C677T polymorphisms and ASD risk in the five genetic models: allelic (*P*_B_ = 0.029, *P*_E_ = 0.017), heterozygote (*P*_B_ = 0.017, *P*_E_ = 0.008), homozygote (*P*_B_ = 0.048, *P*_E_ = 0.053), dominant: (*P*_B_ = 0.021, *P*_E_ = 0.010), and recessive models (*P*_B_ = 0.033, *P*_E_ = 0.053). However, publication bias was detected among the studies on the correlation between *MTHFR* A1298C polymorphisms and ASD risk in the following genetic models: allelic (*P*_B_ = 0.210, *P*_E_ = 0.010), heterozygote (*P*_B_ = 0.001, *P*_E_ = 0.049), homozygote (*P*_B_ = 0.025, *P*_E_ = 0.152), dominant (*P*_B_ = 0.000, *P*_E_ = 0.049), and recessive models (*P*_B_ = 0.081, *P*_E_ = 0.152) (Tables 3 and 4, Fig. 4).

## Discussion

Relevant and up to date literature published prior to January 26, 2020 were selected for examining the correlation between *MTHFR* polymorphism (C677T and A1298C) and ASD risk in this meta-analysis. The findings of this study exhibit that *MTHFR* C677T polymorphism is a susceptibility factor of ASD, but *MTHFR* A1298C polymorphism is not linked with ASD susceptibility.

Several meta-analytic studies on the correlation between C677T polymorphism of *MTHFR* and ASD risk have been conducted. Frustaci et al. [24] studied six articles [22, 27, 28, 35, 43, 44], which consisted of 877 cases and 939 controls, mainly Caucasians, and found a remarkable correlation between C677T polymorphism of *MTHFR* and ASD risk [24]. Pu et al. [25] investigated eight articles [9, 18, 22, 27, 28, 31, 35, 43] involving 1672 cases and 6760 controls, also mainly Caucasians, evidenced a significant risk on the T allele mutation of *MTHFR* C677T in ASD [25]. Rai et al. [26] investigated 1978 cases and 7257 controls (Caucasians: 1355 cases and 6460 controls; Asians: 623 cases and 797 controls) in 13 studies [18, 22, 27, 28, 31, 33, 35, 43, 44, 46, 48, 50] and found that C677T polymorphism of *MTHFR* is a risk factor for ASD susceptibility as well [26]. Similarly, the current meta-analysis enrolled 2609 cases and 7496 controls (Caucasian: 1786 cases and 6499 controls, Asian: 823 cases and 997 controls) from 15 selected literature [9, 18, 22, 26–28, 31, 32, 33, 35, 43, 47, 48, 50], further confirmed the association between C677T polymorphism of *MTHFR* and ASD susceptibility.

A previous meta-analysis, conducted on the correlation between A1298C polymorphism of *MTHFR* and ASD risk [25] (included five literatures; 1470 cases and 1060 controls; Caucasians: 1332 cases and 922 controls, Asians: 138 cases and 138 controls, respectively) [18, 22, 35, 43, 44] reported that A1298C polymorphism of *MTHFR* is remarkably linked to reduced ASD risk but only in the recessive model [25].

In the present meta-analysis, eight of the selected articles [18, 22, 32, 35, 36, 43, 44, 47, 50] had enrolled 1961 cases and 1652 controls (Caucasians: 1387 cases and 991 controls, Asians: 574 cases and 661 controls), and it was recognized that A1298C polymorphism of *MTHFR* was not correlated with ASD susceptibility. However, Khalil et al. (42 cases and 48 controls) [49] and El-Baz et al. (31 cases and 39 controls) [32] revealed that *MTHFR* A1298C polymorphism represented a risk factor in association with ASD. This disagreement may be caused by small samples in the study.

There are several limitations for this study. First, the subgroup analyses of environmental risk factors, sex, and gene-environment interactions were not performed owing to insufficient information. Second, this meta-analysis was mainly focused on Caucasians and Asians, thus limiting the generalization of the findings to other ethnicities. Third, in agreement with the findings of Frustaci et al. [24], Pu et al. [25] and Rai et al. [26], heterogeneity exists in this exploration. Fourth, publication bias was found in the association between *MTHFR* A1298C polymorphisms and ASD risk.

## Conclusion

To conclude, this meta-analysis confirms that C677T polymorphism of *MTHFR* is remarkably linked with ASD risk. Nevertheless, the findings agree that the A1298C polymorphism of *MTHFR* is not significantly correlated with ASD. Exploring gene-gene and gene-environment interactions could throw more light on the genetic link between *MTHFR* variants and ASD risk.

## Supplementary information

**Additional file 1 **: **Supplement file 1.** Search strategy: For this meta-analysis, a total of 15 manuscripts published up to January 26, 2020, were selected from PubMed, Google Scholar, Medline, WangFang, and CNKI databases using search terms “MTHFR” OR “methylenetetrahydrofolate reductase” AND “ASD” OR “Autism Spectrum Disorders” OR “Autism” AND “polymorphism” OR “susceptibility” OR “C677T” OR “A1298C”.

## Data Availability

The datasets used and/or analysed during the current study are available from the corresponding author on reasonable request.

## Abbreviations

ASD: Autism spectrum disorder
MTHFR: Methylenetetrahydrofolate reductase
SNPs: Single nucleotide polymorphisms
HWE: Hardy-Weinberg equilibrium
OR: Odds ratio
CI: Confidence interval
AIC: Akaike’s information criterion
LD: Linkage disequilibrium
