# Supplementary material for: Association between MTHFR C677T/A1298C and susceptibility to autism spectrum disorders: a meta-analysis
Source: BMC Pediatr. 2020 Sep 24;20:449. doi: 10.1186/s12887-020-02330-3 (PMC7517654; doi:10.1186/s12887-020-02330-3)
Supplement: Supplementary file 1 — Additional file 1 : Supplement file 1. Search strategy: For this meta-analysis, a total of 15 manuscripts published up to January 26, 2020, were selected from PubMed, Google Scholar, Medline, WangFang, and CNKI databases using search terms “MTHFR” OR “methylenetetrahydrofolate reductase” AND “ASD” OR “Autism Spectrum Disorders” OR “Autism” AND “polymorphism” OR “susceptibility” OR “C677T” OR “A1298C”. [file 12887_2020_2330_MOESM1_ESM.docx]

Supplement file 1. Search strategy

Search strategy: For this meta-analysis, a total of 15 manuscripts published up to January 26, 2020, were selected from PubMed, Google Scholar, Medline, WangFang, and CNKI databases using search terms “MTHFR” OR “methylenetetrahydrofolate reductase” AND “ASD” OR “Autism Spectrum Disorders” OR “Autism” AND “polymorphism” OR “susceptibility” OR “C677T” OR “A1298C”.
